# Metastatic small cell lung cancer presenting as acute appendicitis: a case report

**DOI:** 10.1093/jscr/rjad101

**Published:** 2023-03-07

**Authors:** Louis Connell, Ziqin Ng, Wei Zhong Tai, Ben Allanson

**Affiliations:** Acute Surgical Unit, Sir Charles Gairdner Hospital Perth, Western Australia, Australia; Acute Surgical Unit, Sir Charles Gairdner Hospital Perth, Western Australia, Australia; Acute Surgical Unit, Sir Charles Gairdner Hospital Perth, Western Australia, Australia; Acute Surgical Unit, Sir Charles Gairdner Hospital Perth, Western Australia, Australia

## Abstract

We present a case of extensive stage small cell lung cancer presenting as perforated appendicitis secondary to an appendiceal metastasis. This is a rare presentation with only six reported cases in the literature. Surgeons must be aware of unusual causes for perforated appendicitis as in our case the prognosis can be dire. A 60-year-old man presented with an acute abdomen and septic shock. Urgent laparotomy and a subtotal colectomy were performed. Further imaging suggested the malignancy was secondary to a primary lung cancer. Histopathology demonstrated a ruptured small cell neuroendocrine carcinoma in the appendix with thyroid transcription factor 1 positive immunohistochemistry. Unfortunately, the patient deteriorated due to respiratory compromise and was palliated day six postoperatively. Surgeons should consider a broad differential diagnosis for the cause of acute perforated appendicitis as this can rarely be due to a secondary metastatic deposit from a widespread malignant process.

## INTRODUCTION

Lung cancer remains the leading cause of cancer death in Australia, comprising 9% of all new cancer diagnoses and an estimated 17% of cancer related deaths in 2022 [1]. Neuroendocrine tumours comprise 25% of all primary lung carcinomas, with the most common type of neuroendocrine, poorly differentiated tumours that have distant metastasis on presentation in the majority of cases [2]. Although widespread metastasis is common, metastasis to the appendix is rare, with only six cases of small cell lung carcinoma with appendiceal metastasis identified in the literature [3]. We present a case of a 60-year-old male with heavy smoking history who presented with appendiceal metastasis causing acute perforated appendicitis.

This case report has been produced in keeping with the SCARE criteria [4].

**Figure 1:**
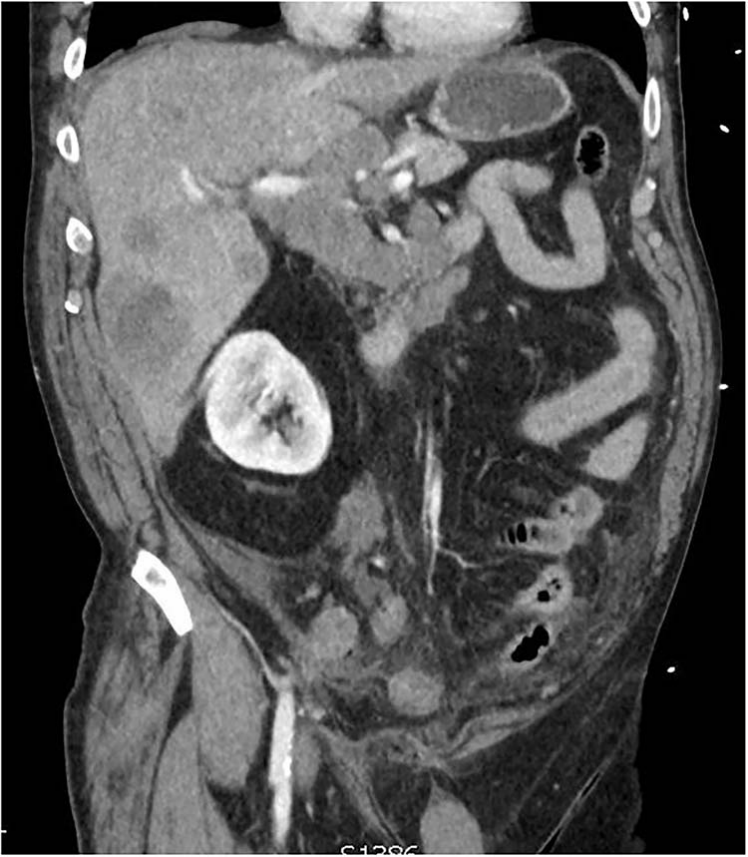
CT of the abdomen from admission demonstrating hepatic lesions, lymphadenopathy and a perforated appendix.

## PRESENTATION OF CASE

A 60-year-old male presented with 4-h history of acute right lower abdominal pain. He had a history of emphysema, hypertension, heavy cigarette smoking and prostate cancer in remission. On presentation, he was septic with tachycardia (110/min), hypotensive with systolic blood pressure of 90 mmhg and febrile (38.5°C). Abdominal examination revealed peritonism in the right lower quadrant. Computed tomography (CT) scan was performed demonstrating a perforated appendix with a 51 × 31 × 26 mm collection and surrounding fat stranding. CT scan also demonstrated innumerable hepatic nodules, mural thickening of the caecum and sigmoid colon as well as marked mesenteric, para-aortic, portocaval lymphadenopathy and pulmonary consolidation/collapse in the right lower lobe (Fig. 1). Patient underwent an exploratory laparotomy. Intra-operative findings were of a perforated appendix with four quadrant purulent contamination and a thickened and woody sigmoid colon. A subtotal colectomy with end ileostomy was performed. Post operatively the patient remained intubated and ventilated.

**Figure 2:**
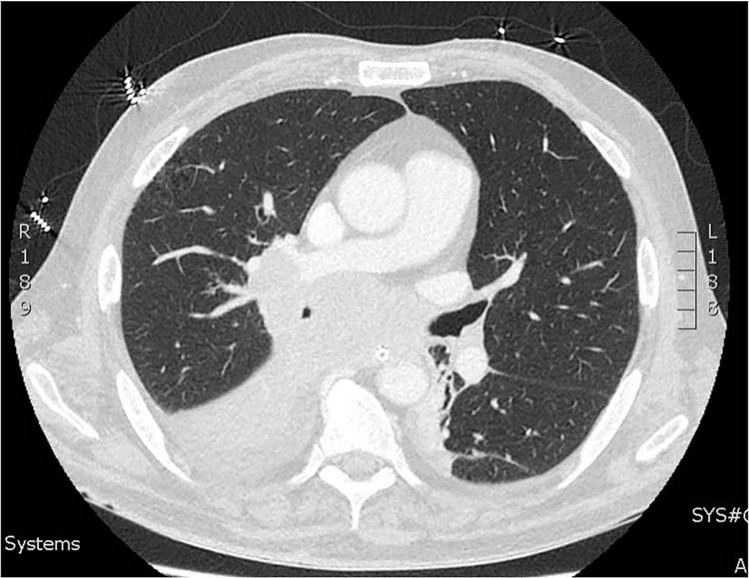
CT of the chest from day two admission demonstrating soft tissue mass enveloping the right bronchi.

On day two of post laparotomy, a CT chest was performed for disease staging (Fig. 2). This study demonstrated a bulky multi-station mediastinal lymphadenopathy contiguous with soft tissue thickening enveloping the right lower lobe and middle lobe bronchi, suggestive of a primary lung cancer. Extubation was attempted on day three post laparotomy, however, the patient desaturated and was re-intubated and ventilated. Following family discussion regarding the poor prognosis care was withdrawn day six and the patient passed away.

**Figure 3:**
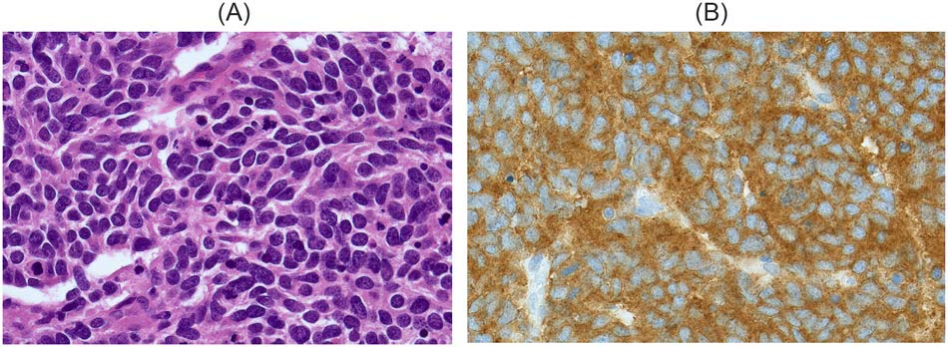
**A**: A high-power field (60×) demonstrating lesional cells with a small cell morphology. They are round to oval shaped with scant cytoplasm and poorly defined cell borders, with hyperchromatic nuclei and occasional inconspicuous nucleoli. There is nuclear moulding and frequent mitotic figures. **B**: Immunohistochemistry on tumour cells is positive for synaptophysin, chromogranin A and CD56. Metastatic Merkel cell carcinoma is considered less likely as there is no cytoplasmic CK20 staining. Tumour cells show nuclear staining for TTF1, which is consistent with but nonspecific for metastatic small cell carcinoma of lung.

Histopathology of the subtotal colectomy results demonstrated ruptured small cell neuroendocrine carcinoma in the appendix and chronic sigmoid diverticular disease with fibrosis (Fig. 3). Immunohistochemistry staining was thyroid transcription factor 1 (TTF1) positive, with p53 over expression, loss of RB1, CD56 positive and negative for CXD2. The same cells were demonstrated in cytology from bronchoalveolar lavage, consistent with high grade metastatic small cell neuroendocrine carcinoma.

## DISCUSSION

The median survival for metastatic small cell lung carcinoma is 9–11 months and 5-year survival is 7% [5, 6]. With a shorter median survival in a cohort of 366 lung cancer patients with gastrointestinal metastasis being ˂3 months [7]. Lung origin small cell neuroendocrine carcinomas tend to metastasise, in descending frequency, to the liver, bone and brain [8]. Clinically symptomatic metastasis to the bowel from lung cancer is rare and it is rarer still to metastasise to the appendix [9, 10]. The incidence of gastrointestinal metastasis from lung cancer is ˂2% in clinical studies, however up to 14% in autopsy studies, indicating undetected asymptomatic spread in a large portion of cases with advanced lung cancer [9]. The likely route of spread of lung cancer to the gastrointestinal tract is through haematogenous spread and via ingested sputum [9, 10]. Taira *et al.* demonstrated one case of metastasis to the appendix of 2066 cases of lung cancer in a retrospective study, the appendix metastasis presented with acute appendicitis [11]. A recently published literature review by Callum *et al.* identified 12 cases of lung cancer with appendiceal metastasis of which half were small cell carcinoma and 10 of 12 presented with perforated appendicitis [4]. CT scan alone has a 72% sensitivity for detection of lung cancer metastasis to the gastrointestinal tract, whilst positron emission tomography-CT is more likely to detect occult metastases [7]. Indeed lung cancer involving the gastrointestinal tract has no differentiating features from primary gastrointestinal cancer on imaging and so diagnosis needs to be made considering the imaging, histopathology and immunohistochemistry [12]. Although neuroendocrine tumours can arise in any body region 95% of all small cell carcinomas are lung origin and thus a small cell carcinoma presenting in any body region with should prompt investigation for a lung primary tumour [2].

Final differentiation of lung origin from gastrointestinal neuroendocrine tumour relies on immunohistochemistry and complete imaging studies. In our case positive immunostaining for TTF1 with negative staining for Caudal-type Homeobox 2 (CDX2) lead us to suspect a lung origin tumour as TTF-1 is highly sensitive for lung origin carcinomas^13.^ In contrast primary colorectal tumours tend to demonstrate the opposite pattern being positive for CDX2 and negative for TTF1, with only 2.5% of primary colon cancers being positive for TTF1 [12, 13]. Therefore, in the majority of cases, TTF1 can be confidently used to differentiate bowel from lung origin adenocarcinomas [12–14]. However, the interpretation of immunohistochemistry for high grade small cell carcinomas is more nuanced as for small cell carcinomas TTF1 is sensitive for lung origin, but not specific^14.^ Hence, in our case final determination of the primary was based on the combination of the imaging findings, histopathology and supported by the immunohistochemistry.

There is insufficient clinical data to indicate clearly the optimal management of metastatic lung cancer presenting with acute appendicitis. However, where there is evidence of perforation and sepsis intervention is indicated. Further to this a retrospective analysis of 366 cases of lung cancer with gastrointestinal metastasis demonstrated abdominal surgical intervention to be a positive prognostic factor, although this may have been confounded by selection bias [7]. We suggest intervention where required for sepsis control or symptom relief.

## CONCLUSION

This report presented a rare case of metastatic small cell lung carcinoma presenting as acute appendicitis secondary to an appendiceal metastasis. Surgeons should consider a broad differential diagnosis for the cause of acute perforated appendicitis as this can rarely be due to a secondary metastatic deposit.

## CONFLICT OF INTEREST STATEMENT

None declared.

## FUNDING

None.
